# Predicting adolescent psychopathology from early life factors: A machine learning tutorial

**DOI:** 10.1016/j.gloepi.2024.100161

**Published:** 2024-08-29

**Authors:** Faizaan Siddique, Brian K. Lee

**Affiliations:** a Department of Epidemiology and Biostatistics, School of Public Health, Drexel University, Philadelphia, PA, United States of America; b Conestoga High School, Berwyn, PA, United States of America; c Department of Global Public Health, Karolinska Institutet, Stockholm, Sweden

**Keywords:** Adolescent, Child, Pregnancy, Mental disorders, Machine learning, Risk prediction

## Abstract

**Objective:**

The successful implementation and interpretation of machine learning (ML) models in epidemiological studies can be challenging without an extensive programming background. We provide a didactic example of machine learning for risk prediction in this study by determining whether early life factors could be useful for predicting adolescent psychopathology.

**Methods:**

In total, 9643 adolescents ages 9–10 from the Adolescent Brain and Cognitive Development (ABCD) Study were included in ML analysis to predict high Child Behavior Checklist (CBCL) scores (i.e., t-scores ≥ 60). ML models were constructed using a series of predictor combinations (prenatal, family history, sociodemographic) across 5 different algorithms. We assessed ML performance through sensitivity, specificity, F1-score, and area under the curve (AUC) metrics.

**Results:**

A total of 1267 adolescents (13.1 %) were found to have high CBCL scores. **The best performing algorithms were elastic net and gradient boosted trees. The best performing elastic net models included prenatal and family history factors (Sensitivity 0.654, Specificity 0.713; AUC 0.742, F1-score 0.401) and prenatal, family, history, and sociodemographic factors (Sensitivity 0.668, Specificity 0.704; AUC 0.745, F1-score 0.402).** Across all 5 ML algorithms, family history factors (e.g., either parent had nervous breakdowns, trouble holding jobs/fights/police encounters, and counseling for mental issues) and sociodemographic covariates (e.g., maternal age, child's sex, caregiver income and caregiver education) tended to be better predictors of adolescent psychopathology. The most important prenatal predictors were unplanned pregnancy, birth complications, and pregnancy complications.

**Conclusion:**

Our results suggest that inclusion of prenatal, family history, and sociodemographic factors in ML models can generate moderately accurate predictions of adolescent psychopathology. Issues associated with model overfitting, hyperparameter tuning, and system seed setting should be considered throughout model training, testing, and validation. Future early risk predictions models may improve with the inclusion of additional relevant covariates.

## Introduction

Approximately 1 in 6 young adults are diagnosed with a mental health disorder in the US [1,2]. Untreated mental health conditions can have a significant negative impact on quality of life, social interactions, and cognitive function by reducing an individual's confidence in their own abilities [3]. Because adolescence characterizes a phase of crucial cognitive development in humans [4], improving mental health prevention and treatment programs among young populations has been seen as an effective means of boosting overall wellbeing across future generations.

A higher prevalence of mental health disorders have been associated with a variety of genetic and environmental exposures, including prenatal factors, lifestyle changes, family history, and socioeconomic background [[5], [6], [7], [8]]. The relationship between prenatal events and psychiatric illnesses is well-established in the literature because of long-standing neurodevelopmental theories like the Developmental Origins of Health and Disease (DOHaD) hypothesis, which was first introduced by Barker et al. in the 1990s. This concept emphasizes the importance of environmental conditions during the prenatal period of pregnancy, which increase the risk for developing mental health disorders throughout adulthood [[9], [10], [11]]. Meanwhile, others have suggested that a combination of shared familial exposures may contribute to adverse psychiatric outcomes, especially if these conditions affected extended family members [7,12].

Given the existence of potential interactions between individual early life exposures, the cumulative impact of multiple exposures on adolescent cognitive health is less understood. In particular, Roffman et al. previously demonstrated that the aggregation of prenatal risk factors proportionally increases the likelihood for developing mental health disorders [13]. However, family mental health history is important to consider in conjunction with environmental exposures because mental health is highly heritable and risk genes may confer additional vulnerability to environmental exposures [14].

Prior studies have used machine learning (ML) to elucidate the impact of different risk predictors on mental health since it offers both computational power and flexibility [15,16]. While certain ML algorithms may prove to be informative of adverse mental health outcomes [17], most predictive models remain insufficient for clinical use [16,18,19]. The need for advanced knowledge and programming expertise can pose significant barriers to clinical researchers for ML applications [16]. Furthermore, a variety of factors must be considered when training ML models, including limitations associated with model tuning [20], model overfitting [17], small sample populations [21], and model accuracy [[17], [18], [19], [20]].

In light of these advantages and challenges, we aim to provide a general outline for clinical researchers that intend to carry out ML-driven data analyses. We also seek to clarify any common misconceptions and errors that may arise from utilizing ML in a clinical study. We demonstrate its use in this study by predicting adolescent psychopathology through risk profiles containing prenatal, family history, and sociodemographic factors.

## Methods

### ML Overview

With the growing availability of detailed mental health records to researchers, modern ML has enabled analysis of complex research inquiries otherwise unaccounted for by traditional statistical methods [22]. Common frequentist statistical tools, such as regression and hypothesis testing, are susceptible to the “curse of dimensionality” [23,24], where high-dimensional data (i.e., data with lots of features, or variables of interest) is increasingly sparse, which means it is more difficult to uncover underlying data structure without addition of exponentially more data points [25]. While frequentist regression can be ineffective in this situation, ML remains promising for handling large datasets with high nonlinearity and multicollinearity [22,26,27].

An important application of ML is in precision psychiatry, an area of study that has received more attention in recent years [26,28]. Precision psychiatry offers potential for personalized patient care on an individual level, beyond general diagnostic evaluations and clinical criteria traditionally used for discerning mental health disorders. Researchers are hopeful that ML tools may, for example, be able to propose customized treatment plans for different individuals based on their environmental, genetic, and lifestyle-based risk factors [26].

### ML Applications

Most ML techniques may be categorized as either forms of supervised learning or unsupervised learning [26,29]. Supervised learning involves training ML algorithms on labelled data, where the desired outcome has been manually designated by humans so that the final model may perform a specific task or function. For example, one supervised ML application might include predicting disease diagnoses from neuroimaging data after training on a dataset previously classified or labelled as disease/no disease by experts [30,31]. Labelling large datasets can require significant resources and can be time-consuming [31]. Conversely, unsupervised learning allows for training ML algorithms on unlabeled data, where models are prompted to cluster features based on their underlying similarities [29]. Unlike its supervised counterpart, unsupervised learning has no definite measured outcome [31]. Common unsupervised ML applications include clustering diseases into their subtypes, identifying unobserved behavioral dimensions, or developing alternate methods for disease classification [26,31]. Other forms of ML in psychiatry research include semi-supervised learning, which leverages both labelled and unlabeled training data, and reinforcement learning, which balances model reward and punishment to simulate various human behaviors [26,32].

A notable subfield of ML is deep learning (DL), which can enable interpretation of highly complex data patterns from raw features in exchange for greater computational costs [33]. DL applications rely on artificial neural networks (ANNs) and their variants for predicting disease outcomes and have shown great promise in the realm of neuroimaging and image analysis [26,34,35].

### ML Caveats

Despite the numerous benefits that ML offers, there remain important gaps to be addressed that motivate this tutorial. The successful implementation of ML in a study requires careful dataset curation, feature selection, model training, hyperparameter tuning, and model testing. While numerous studies have illustrated ML model performance on testing data across different use cases [18,19,[36], [37], [38]], few studies have attempted to expand upon the thought process required to produce these models from start to finish. Another common drawback of ML algorithms is that they are “black box” [39], meaning that it is often difficult to understand how an algorithm arrived at a prediction. For these reasons, we present the full implementation of a supervised ML approach, with commentary about choices that should be made throughout an ML-driven analysis, to predict adolescent psychopathology from early life factors. To enable interpretability, we include standard logistic regression models as well for comparison.

### Study sample

In practice, the choice of dataset and sample size will vary depending on the research question [22]. The primary goal of this dataset curation step is to gather sufficient data, representative of the target population, such that ML models would be capable of providing unbiased risk predictions without overfitting.

The Adolescent Brain and Cognitive Development (ABCD) Study provides a unique opportunity for this analysis due to the extensive metrics and questionnaires that it provides for a single large cohort of children [40]. In total, the ABCD Study enrolled 11,875 children from ages 9 to 10 across 21 different U.S. sites. De-identified ABCD data are freely available at the National Institute of Mental Health Data Archive.

We made use of baseline data available in the ABCD Study when extracting relevant prenatal events, family history, demographic and household information, and outcome measures. Among the 11,875 participants in the longitudinal ABCD Study cohort, 9643 children were selected for analysis. We excluded 2224 children that had twin siblings due to known effects of clustering across both risk categories [41]. Furthermore, we omitted another 8 participants that did not have valid measures of the primary outcome (CBCL scores, see below, i.e., had missing values in one or more syndrome subscales). General inclusion criteria for the study included children between the ages of 9 and 10 years from the general population that were fluent in English, had no history of major neurological issues or traumatic injuries, and were able to undergo MRI scanning [13,42,43]. The ABCD Study was approved by the Institutional Review Boards at each site and a central IRB (University of California, San Francisco). Written approval was obtained by all parents and all children consented to participation for the study [44,45].

To enhance critical appraisal and assessment of quality, we recommend that scientific reporting guidelines be followed. Here, we conformed to Strengthening the Reporting of Observational Studies in Epidemiology (STROBE). Additionally, because of the prediction modeling employed in this study, we also adhered to Transparent reporting of a multivariable prediction model for individual prognosis or diagnosis (TRIPOD) guidelines for this study [46,47]. STROBE and TRIPOD checklists for the present study are included in the Appendix.

### Adolescent psychopathology

The Child Behavior Checklist (CBCL) was used to measure adolescent mental health outcomes as t-scores. The CBCL questionnaire assesses emotional, behavioral, and social problems in adolescents and consists of 8 syndrome subscales (Anxiety/Depression, Somatic Complaints, Social Problems, Thought Problems, Attention Problems, Rule-breaking Behavior, Aggressive Behavior), 2 larger scales (Internalizing and Externalizing Syndromes), and a total score [48]. The CBCL is a dimensional assessment tool and as such, the score quantifies severity in a continuous fashion. However, risk prediction models most frequently aim to separate individuals into discrete risk categories, such as no/low risk and high risk for a given outcome. Therefore, usage of a continuous score will require choosing clinically relevant cutpoints. Here, we created a binary variable that identified individuals with total t-scores greater than or equal to 60 as having “high” CBCL scores, like previously characterized in the literature as a meaningful threshold for clinically significant psychopathology [13,48,49]. However, we recognize that the psychometric properties of the CBCL, as with any other mental health instrument, will vary according to the sample in which it is employed, and thus recommend that sensitivity analyses explore whether utilizing other cutpoints yield qualitatively different findings.

### Prenatal Exposures

Multiple prenatal exposures may increase the risk for developing mental health disorders later in life. Following the work of Roffman et al. [13], we examined a total of 8 prenatal factors, as assessed by caregiver report in the ABCD *Developmental History Questionnaire* [40]. All exposures were coded as dichotomous variables (present/absent) and are included below:

(1) Had an unplanned pregnancy
(2) Maternal use of tobacco (*before*) knowing about the pregnancy
(3) Maternal use of alcohol (*before*) knowing about the pregnancy
(4) Maternal use of marijuana (*before*) knowing about the pregnancy
(5) Had a Caesarian section
(6) Had pregnancy complications
(7) Had birth complications
(8) Had a pre-term birth

Pregnancy complications were coded as present if at least 1 of 13 predetermined complications were found: severe nausea and vomiting beyond 6th month or weight loss, heavy bleeding requiring bed rest or special treatment, pre-eclampsia, eclampsia, or toxemia, severe gall bladder attack, persistent proteinuria, rubella during first 3 months of pregnancy, severe anemia, urinary tract infection, pregnancy-related diabetes, pregnancy-related high blood pressure, previa, abruptio, or other problems with placenta, accident or injury requiring medical care, and any other conditions requiring medical care [13]. Similarly, birth complications were coded as present if at least 1 of 8 predetermined complications were found: blue at birth, slow heartbeat, did not breathe at first, convulsions, jaundice requiring treatment, required oxygen, required blood transfusion, and Rh incompatibility [13]. Pre-term birth was coded as present if the child was born at least 3 weeks premature (gestational week 37 or earlier).

### Family History

While family history is an excellent indicator of genetic risk, family history can also provide valuable information about the impact of early life household environments on cognitive health. Therefore, we assessed 8 family history related exposures extracted from the ABCD *Family History Assessment* during this study. The following risk factors were dichotomous and were recorded upon report by a caregiver:

(1) Either parent had depression
(2) Either parent had mania
(3) Either parent had visions of others spying/plotting
(4) Either parent had trouble holding jobs, had frequent fights, or police encounters
(5) Either parent with nerves and/or nervous breakdowns
(6) Either parent had counseling for an emotional/mental issue
(7) Either parent had been hospitalized for an emotional/mental issue
(8) Either parent had attempted or committed suicide

### Sociodemographics

A total of 8 variables were used to account for the impact of sociodemographic background on adolescent psychopathology. These variables have been used extensively in previous research [13,50,51], and were obtained from the ABCD *Developmental History Questionnaire, American Community Survey* and *Parent Demographics Survey*. The predictors included:

(1) Mother's age at childbirth (years)
(2) Child's age at time of interview (months)
(3) Child's sex (Male/Female)
(4) Child's race
(5) Child's ethnicity (LatinX/non-LatinX)
(6) Presence of partner with primary caregiver
(7) Total caregiver income
(8) Highest caregiver education

Race was divided into the following 5 groups as previously noted: White, Black, Hispanic, Asian, and Other [52]. Total caregiver income was reported through the following categories: <$25k, 25-<50k, 50-<75 k, 75-<100k, 100-<200k, and 200k+. Highest caregiver education was reported through the following categories: <less than HS, HS, College, Associate Degree, Bachelor's Degree, Masters Degree, Professional School Degree, and Doctoral Degree.

### Statistical analysis

#### Overview

The goals of the analysis were to determine whether prenatal, family history, and sociodemographic risk factors could be used to reliably predict adolescent cognitive outcomes. Specifically, we assessed the likelihood of attaining a high CBCL score using both standard logistic regression and machine learning (ML). We conducted all analyses in R4.2.1. (R Foundation for Statistical Computing).

#### Missing data

Because ML often involves many variables, there is a high likelihood of encountering missing data. Some ML methods may directly handle missing data, for example, a tree-based method that splits based on missingness or not. However, other ML methods do not and as such would require non-missingness in data. Here, the extent of missing data ranged from 8.97 % for average caregiver income to 0.03 % for sex. A total of 23 missing variables received imputation using multiple imputation models contained within the *mice* algorithm in R. Multiple imputation is known to offer significant improvements over listwise deletion (i.e. removal of data points with at least one missing value for the relevant variables being studied) because its introduction of uncertainty can improve model estimates while preserving the order of the original dataset, and deletion of datapoints can introduce bias [53,54]. We imputed continuous and ordinal variables using predictive mean matching, while all other binary variables were imputed using logistic regression as previously shown [13]. In practice, the choice of multiple imputation method does not have a significant impact on performance and is subject to variation across datasets with different missingness patterns [53,55]. We performed 10 iterations of each imputation following existing recommendations in the literature [56]. Following multiple imputation, we conducted bivariate analysis for each relevant exposure against CBCL total scores and syndrome subscores to identify any potential relationships present in the data before regression.

### Standard regression models

Although regression models are generally limited in their capacity to predict outcomes relative to their more powerful ML counterparts [57,58], their results are oftentimes more interpretable and explainable. As a result, regression models are essential to delineating the impact of different early life predictors on mental health outcomes prior to full-fledged ML analysis [59]. In this study, we estimated logistic regression models that predicted the likelihood of obtaining a high CBCL score (i.e., total t-scores ≥ 60), where each prenatal and family history risk factor was included in an unadjusted (no covariates) and adjusted regression model that included sociodemographic covariates. We examined clinically relevant odds ratios to compare the relative effect sizes of each early life predictor.

### ML Algorithm Selection

Choosing an ML algorithm can be complicated, as there are hundreds of possible algorithms to choose from. In supervised ML, models are trained to predict either categorical outcomes (classification) or continuous outcomes (regression) [60]. Depending on this specific task, factors that may influence one's decision of ML algorithms to use may include comparative prediction performance in the validation dataset, computational ease (e.g., processing time and ease of implementation), and what ML algorithms other investigators have used. Here, we implemented 5 different ML algorithms as part of R's *caret* package: bagged CART (classification and regression trees), random forests, gradient boosted trees, neural networks, and elastic net [61]. These algorithms have been previously used in mental health prediction modeling [18,20,62,63], and were easily implementable in caret. Caret is a popular ML framework in R that enables convenient implementation of many of the most commonly used ML algorithms [54]. A detailed description of these supervised ML methods and their overall advantages and disadvantages may be found in (Table 1).

**Table 1:** Description of commonly used ML algorithms, their advantages and disadvantages.

Algorithm	Description	Pros	Cons	Example
Bagged Classification and Regression Trees (CART)	• Aggregation of decision trees formed on independent bootstrap samples (i.e., random rows of observations sampled repeatedly from the training dataset, with replacement) • **All features** considered for recursive binary splitting (i.e., categorizing data based on binary conditions (nodes) to form a decision tree with branches) • Tree contributions collectively averaged to generate final prediction • Ensemble approach	• Robust to changes in training data • Lower variance than single CART • Suitable for reducing overfitting • Works with missing data (like CART)	• Harder to interpret than CART • Generally lower performing than random forests • Risk of correlated trees increasing bias with select few dominant predictors • More sensitive to class imbalance than boosted trees	Lin et al. [104]
Random Forests	• Aggregation of decision trees formed on independent bootstrap samples • **Random subset of features** considered for recursive binary splitting • Tree contributions collectively averaged to generate final prediction • Ensemble approach	• Robust to changes in training data • Lower variance than CART and Bagged CART • Suitable for reducing overfitting AND risk of correlated trees • Works with missing data (like CART) • Among highest performing models in the literature	• Harder to interpret than CART • Computationally expensive and slow • More sensitive to class imbalance than boosted trees	Li et al. [105]
Gradient Boosted Trees	• Sequential aggregation of shallow decision trees (i.e., trees with few branches, or “weak learners”) formed on entire training dataset • Each subsequent tree in chain optimized to improve upon errors of previous models via gradient descent • Tree contributions collectively combined to generate final prediction • Ensemble approach	• Lower bias than Random Forests in low performance tasks • Suitable for predicting rare events • Among highest performing models in the literature	• Hard to interpret • More prone to overfitting than random forests • Computationally expensive and slow	Ali et al. [106]
Neural Networks	• Interconnected network of neurons (nodes) designed to mirror human cognitive abilities • Layers consisting of weights and biases optimized during training via backpropagation • Information passed from input layer through the network to the output layer (“feed-forward”) to generate final prediction • Foundation for modern DL methods	• Highly flexible with many variants (ANNs, CNNs, RNNs, etc.) • Architecture readily modifiable by user based on specific use case • Suitable for real-time prediction • Among highest performing models in the literature	• Hard to interpret • Computationally expensive and slow, depending on architecture • Susceptible to overfitting (too shallow networks or too many training iterations) or underfitting (too deep networks causing vanishing gradient problem)	Uyulan et al. [107]
Elastic Net	• Regularization of existing regression or ML methods (i.e., adding another penalty term to traditional loss function) • Hybrid of L1 (lasso) and L2 (ridge) regression	• Highly suitable for feature selection and handling multicollinearity • Easier to interpret than other ML methods • Overcomes weaknesses of both lasso and ridge regression • Versatile application	• More computationally expensive and susceptible to overfitting than lasso or ridge regression • May lack predictive power for highly complex problems	Kim et al. [108]

### Preprocessing

For numeric data types (i.e., variables measuring a quantitative value), two preprocessing methods may be applicable: data standardization, or data normalization. Both techniques are appropriate for preventing ML algorithms from unfairly prioritizing numeric variables with larger scales due to their greater perceived effect sizes. While data standardization scales all values of a variable to follow a distribution with mean 0 and standard deviation 1, normalization scales these values to a new specified range, often much smaller than the original.

Categorical data types (nominal, ordinal, binary) should be encoded into numeric form during preprocessing [64]. Since nominal variables contain categories that lack any particular order, one-hot encoding (also known as dummy coding) is the optimal choice for ensuring ML models do not derive ordinal significance from the new dummy labels [65]. In one-hot encoding, each category in a variable receives a new binary feature, so that any data point that falls under a given group has value one for its own binary feature, and zero across all the other binary features corresponding to the same categorical variable. Conversely, ordinal variables contain ordered categories and may be preprocessed with label encoding, or the assignment of categories to numbers in a particular order. Binary variables may receive label encoding if the raw data originally consisted of strings of text instead of zeros and ones.

During preprocessing, one should try to minimize the number of added features by avoiding redundancy (e.g., implementing one-hot encoding for binary variables) and high dimensionality (e.g., including extremely rare event categories as binary features). This improves ML model interpretability by reducing the impact of elevated multicollinearity between newly encoded category features [65].

Here, we employed ordinal and binary predictors with label encoding to predict the probability that an adolescent would have a high CBCL t-score (≥ 60) (classification).

### Feature Selection

For each ML algorithm, variables were split into 3 different categories: prenatal, family history, and sociodemographic risk factors. We then evaluated the predictive performance of each individual category; all pairs of categories; and all categories together. This study design allows for comparative assessment of ML performance based on different sets of predictors in addition to individual comparison of the predictors themselves.

We also demonstrate an alternative ML-driven approach to feature selection through the elastic net model. This regularization technique merges the strengths of Lasso (L1) and Ridge (L2) regression with the inclusion of two tuning parameters, lambda (which controls the strength of the regularization), and alpha (which balances L1 and L2 contributions). This hybrid design allows elastic net to automatically select the most meaningful predictors given to the model without prior specification. Since our outcome of interest is dichotomous (high CBCL or not), the elastic net model is effectively a form of penalized logistic regression.

### Data Partitioning and Resampling

Data was randomly split into 80 % training and 20 % testing–in other words, models are developed on 80 % of the data and performance is tested on the remaining 20 % of the data. The relative sizes of the training and testing datasets provide protection against the common danger of model overfitting in prediction modeling, where a prediction model hypersensitive to noise within training data performs significantly better on known training samples while failing to generalize its results on new testing samples [66]. The size of the testing dataset may vary depending on the problem, but in most cases training data should comprise at least 70 % of the data split [67]. Our final ML models were both trained and tested using a single specified random seed to enhance reproducibility [68].

We considered the following resampling methods when performing model validation: k-fold cross-validation (k-fold CV), leave-one-out cross-validation (LOO-CV), and bootstrap validation [69]. Resampling further combats model overfitting because the model is trained on multiple different training samples that each randomly differ from the true population, thus improving its generalizability by increasing the amount of relevant information the model can extract from training data. For example, in k-fold CV, a given training dataset is divided into k partitions of equal size. Then, k-1 subsets (or folds) are used to fit the ML model, with the remaining unused fold set aside for validation, and the process is repeated over all k possible combinations of k-1 folds [69,70]. This method enables effective usage of the full training dataset, resulting in prediction estimates that are more unbiased and less variable. For smaller datasets with n observations, LOO-CV (the equivalent procedure of k-fold CV when k = n) can be more unbiased than traditional CV [69]. However, since each validation fold consists of only 1 observation, LOO-CV also has high variance, and this comes with the cost of greater computational power needed for larger n [69,70]. While k-fold CV samples without replacement to create non-overlapping folds, bootstrap validation samples with replacement, fitting ML models on k independent random samples of training data. The value of k used for bootstrap validation is much larger than that used for CV (e.g., k = 100, k = 1000, etc.), and this number does not have an explicit upper bound [69]. By the Law of Large Numbers, bootstrap validation is helpful for determining the empirical distribution of a diverse range of model performance metrics, motivating its application in many scenarios [71,72]. Since bootstrap samples can include repeat observations, CV is advantageous when discerning a model's generalizability on unseen data is a main priority.

Since our training dataset was relatively large, we opted to use 10-fold repeated CV. Repeated CV is a simple yet robust extension of traditional CV, as the entire partitioning, training, and validation procedure is repeated a specified number of times [70]. In particular, the number k = 10 was chosen to improve consistency in model performance estimates while still fully leveraging the data for the analysis [69].

### Model Tuning

During ML model training, performance is optimized by a series of hyperparameters, or parameters that govern an algorithm's configuration for a specific task [73]. The optimization of hyperparameters is known as tuning, and these parameters can be either automatically deduced by the computer (automatic grid search) or manually deduced by the user through heuristics (manual grid search). We identified the optimal combination of hyperparameters by employing both manual and automatic grid searches of the parameter space. Due to the variety of possible modifiable hyperparameters for each ML algorithm (e.g. mtry, shrinkage, interaction depth) [74], the choice of tuning often depends on the amount of time and computational power at a researcher's disposal. For example, while manual tuning is usually less computationally expensive and preferrable when the optimal hyperparameters have been discerned through prior knowledge or trial and error, automatic tuning is favorable when researchers wish to test all possible combinations of available hyperparameters.

An important distinction should be drawn between the optimization of ML model parameters and hyperparameters. Unlike hyperparameters, model parameters are internal to an ML architecture and cannot be modified directly by the user. For instance, the model parameters of a neural network consist of weights and biases that are initialized randomly and approach their optimal values throughout training. The optimization of model parameters is accomplished by finding the local minimum for a loss function, which measures the prediction error of an ML model. Common loss functions include cross entropy for classification ML problems and mean squared error (MSE) for regression problems [75]. Tuning hyperparameters should be chosen to promote successful convergence of model parameters that can achieve the best possible performance on a given dataset.

### Model Validation

We report model performance using a variety of evaluation metrics as previously advised [76,77], including sensitivity, specificity, and F1-scores. The choice of metric typically depends on the type of analysis and intended ML use case. For example, sensitivity (or recall; true positive rate) measures the percentage of positive cases that are correctly classified as positive by the predictive model, making it a meaningful metric for depicting diagnostic ability. Meanwhile, specificity (or true negative rate) measures the percentage of negative cases are correctly classified as negative by the predictive model, so it should be reported when maximizing screening ability is a priority. For determining holistic model performance, the F1-score is a useful hybrid metric that combines precision (or positive predictive value; the percentage of positive predictions that are true positive cases) and recall by computing their harmonic mean [77]. The F1-score is also known as the Dice similarity coefficient in ML-based medical image analysis and remains one of the most used indicators of computer vision model performance [78]. For imbalanced data, the F1-score is especially proficient at recording the balance in performance between minimization of false negatives (high recall) and false positives (high precision) as a single number [77]. Other evaluation metrics include, but are not limited to, Cohen's kappa, Matthews Correlation Coefficient (MCC), and Intersection over Union (IoU) [78,79].

Due to known issues associated with setting default classification thresholds at 0.5 for imbalanced data [80], we separately determined the optimal threshold using receiver operator characteristic (RoC) curves generated by the *cutpointr* package to maximize the Youden Index across each model [81,82]. The Youden Index is one minus the sum of sensitivity and specificity, so maximizing this metric effectively combines both components of the RoC curve (sensitivity and 1-specificity) to select the best performing model [79,83]. Other alternatives include maximizing the F1-score and overall accuracy, although these metrics have been previously shown to be not as effective for threshold optimization [79,81]. The area under the RoC curve (AUC) was also extracted to compare ML model performance on the testing dataset, although it is important to note that this number remains constant regardless of the classification threshold selected [79].

To help with ML model interpretability, we also show feature importance plots for models from each ML algorithm. Briefly, feature importance refers to a score calculated during the modeling process that measures the effect it has on the model. For predictive modeling, feature importance is useful in terms of depicting the relative importance of predictors with respect to each other.

## Results

Summary statistics for the ABCD Study cohort (*N* = 9643) are shown in (Table 2). Participants across both groups had similar demographics with a slightly higher percentage of males having high CBCL scores (60.3 % high CBCL, 51.4 % normal CBCL). The high CBCL score group (*N* = 1267) consisted of fewer White and Asian individuals (44.6 % vs 49.9 %; 0.9 % vs 2.7 %) and more individuals from Black and other racial backgrounds (18.2 % vs 14.9 %; 13.9 % vs 10.2 %). Adolescents with high CBCL scores were less likely to have their primary caregiver accompanied by a partner (71.4 % vs 81.1 %), a higher total caregiver income (25.5 % vs 39.7 %) and higher caregiver education (81.5 % vs 84.3 %). Additionally, adolescents in the high CBCL group generally reported a higher number of risk exposures relative to their normal counterparts, excluding Caesarian section and pre-term birth where distributions were approximately similar.

**Table 2:** Characteristics of adolescent population (N = 9643) assessed in study. Figures are numbers (percentage) unless otherwise denoted.

Category	Characteristics		Overall sample (N = 9643)	CBCL t-score ≥ 60 (*N* = 1267)	CBCL t-score < 60 (*N* = 8376)
Sociodemographic	Age (months)⁎		118.5 (7.6)	118.5 (7.5)	118.6 (7.6)
	Mother's age (years)⁎		29.0 (6.3)	27.6 (6.7)	29.2 (6.2)
	Sex (Male)		5067 (52.5)	764 (60.3)	4303 (51.4)
	Race	White	4747 (49.2)	565 (44.6)	4182 (49.9)
		Black	1477 (15.3)	231 (18.2)	1246 (14.9)
		Hispanic	2148 (22.3)	283 (22.3)	1865 (22.3)
		Asian	242 (2.5)	12 (0.9)	230 (2.7)
		Other	1029 (10.7)	176 (13.9)	853 (10.2)
	Ethnicity (LatinX)		2160 (22.4)	285 (22.5)	1875 (22.4)
	Presence of partner		7696 (79.8)	905 (71.4)	6791 (81.1)
	Total caregiver income (100 k+)		3651 (37.9)	323 (25.5)	3328 (39.7)
	Highest caregiver education (college or higher)		8093 (83.9)	1032 (81.5)	7061 (84.3)
Prenatal	Unplanned pregnancy		3977 (41.2)	708 (55.9)	3269 (39.0)
	Early use of tobacco		1385 (14.4)	350 (27.6)	1035 (12.4)
	Early use of alcohol		2588 (26.8)	411 (32.4)	2177 (26.0)
	Early use of marijuana		672 (7.0)	188 (14.8)	484 (5.8)
	Caesarian section		2963 (30.7)	394 (31.1)	2569 (30.7)
	Pregnancy complications		3784 (39.2)	668 (52.7)	3116 (37.2)
	Birth complications		2291 (23.8)	420 (33.1)	1871 (22.3)
	Pre-term birth		535 (5.5)	93 (7.3)	442 (5.3)
Family History	Depression		1179 (12.2)	318 (25.1)	861 (10.3)
	Mania		549 (5.7)	171 (13.5)	378 (4.5)
	Visions of others spying/plotting		248 (2.6)	85 (6.7)	163 (1.9)
	Trouble holding jobs/fights		1404 (14.6)	404 (31.9)	1000 (11.9)
	Nervous breakdowns		1261 (13.1)	366 (28.9)	895 (10.7)
	Counseling for emotional/mental issue		3974 (41.2)	818 (64.6)	3156 (37.7)
	Hospitalization for emotional/mental issue		950 (9.9)	287 (22.7)	663 (7.9)
	Suicide attempt		629 (6.5)	197 (15.6)	432 (5.2)

⁎ =continuous variable denoted as mean (standard deviation).

### Regression models

Throughout both unadjusted and fully adjusted logistic regression models, all exposures independently increased the odds of having CBCL scores ≥ 60 (Table 3). Most prenatal and family history predictors significantly increased the odds of having high CBCL scores in unadjusted models but exhibited lower odds in adjusted models including covariates. Meanwhile, pregnancy complications, birth complications, pre-term birth, and counseling for an emotional/mental issue had similar odds of high CBCL scores across both types of models. Parental visions of spying or plotting, trouble holding jobs/fights, and hospitalization for an emotional/mental issue had the greatest odds for developing clinically significant psychopathology in unadjusted logistic regression models (OR 3.62, 95 % CI 2.76 to 4.73; OR 3.45, 95 % CI 3.01 to 3.95; OR 3.41, 95 % CI 2.92 to 3.97). A family history of counseling for an emotional/mental issue and nervous breakdowns were most strongly associated with high CBCL scores when accounting for covariates (OR 3.17, 95 % CI 2.79 to 3.62; OR 2.98, 95 % CI 2.58 to 3.45).

**Table 3:** Logistic regression model results from 16 minimally and 16 fully adjusted models pooling both sets of exposures together (recorded as odds ratios (CI) for high CBCL, bold if *p* < 0.05).

Categories	Exposures	High CBCL	
		Unadjusted	Adjusted⁎
Prenatal	Unplanned pregnancy	**1.98 (1.76 to 2.23)**	**1.58 (1.38 to 1.81)**
	Early use of tobacco	**2.71 (2.35 to 3.11)**	**2.13 (1.83 to 2.47)**
	Early use of alcohol	**1.37 (1.20 to 1.55)**	**1.60 (1.40 to 1.83)**
	Early use of marijuana	**2.84 (2.37 to 3.40)**	**2.27 (1.88 to 2.74)**
	Caesarian section	1.02 (0.90 to 1.16)	1.04 (0.91 to 1.18)
	Pregnancy complications	**1.88 (1.67 to 2.12)**	**1.72 (1.53 to 1.95)**
	Birth complications	**1.72 (1.52 to 1.96)**	**1.70 (1.49 to 1.94)**
	Pre-term birth	**1.42 (1.12 to 1.78)**	**1.36 (1.07 to 1.72)**
Family History	Depression	**2.92 (2.53 to 3.38)**	**2.32 (1.99 to 2.71)**
	Mania	**3.30 (2.72 to 3.99)**	**2.71 (2.22 to 3.29)**
	Visions of others spying/plotting	**3.62 (2.76 to 4.73)**	**2.78 (2.09 to 3.67)**
	Trouble holding jobs/fights	**3.45 (3.01 to 3.95)**	**2.71 (2.33 to 3.14)**
	Nervous breakdowns	**3.40 (2.95 to 3.90)**	**2.98 (2.58 to 3.45)**
	Counseling for emotional/mental issue	**3.01 (2.67 to 3.41)**	**3.17 (2.79 to 3.62)**
	Hospitalization for emotional/mental issue	**3.41 (2.92 to 3.97)**	**2.78 (2.37 to 3.26)**
	Suicide attempt	**3.39 (2.82 to 4.05)**	**2.66 (2.20 to 3.20)**

⁎ Adjusted for sociodemographic covariates (child's age, sex, race, ethnicity, mother's age, presence of partner, total caregiver income, and highest caregiver education).

### Machine learning models

Classification ML model performance on testing data varied across all 5 ML algorithms based on the optimal cutpoint chosen for maximizing Youden's Index (Table 4). Elastic net generated the highest performing models during ML analysis when pooling all risk predictors, with sensitivities of 0.668 and 0.654 and specificities of 0.704 and 0.713 for with and without sociodemographic predictors respectively (AUC 0.745 and 0.742; F1-score 0.402 and 0.401). Gradient boosted trees exhibited similar high performance across models with analogous predictor combinations (AUC 0.723 and 0.734; F1-score 0.390 and 0.393). Model performance gradually improved as more predictors were added to the bagged CART and random forests models, with their highest performing models including all 3 sets of predictors (sensitivity 0.630 and 0.630; specificity 0.601 and 0.669; AUC 0.658 and 0.695). On the contrary, gradient boosted trees, neural network, and elastic net model performance was more consistent across all combinations of predictors. A tradeoff between increased sensitivity and decreased specificity was observed as ML models incorporated more predictor variables. Bagged CART exhibited the largest variation in performance with the lowest sensitivity of 0.134 (AUC 0.541) for the prenatal ML model.

**Table 4:** Test set performance of machine learning classification models using different combinations of predictors (recorded as (sensitivity, specificity; F1-score, AUC)).

Predictor Combinations	Bagged CART	Random Forests	Gradient Boosted Trees	Neural Networks	Elastic Net
Prenatal	(0.134, 0.949; 0.188, 0.541)	(0.223, 0.903; 0.252, 0.567)	(0.627, 0.597; 0.323, 0.660)	(0.627, 0.600; 0.324, 0.652)	(0.623, 0.649; 0.347, 0.675)
Family History	(0.212, 0.936; 0.270, 0.574)	(0.264, 0.914; 0.303, 0.600)	(0.442, 0.830; 0.369, 0.696)	(0.497, 0.778; 0.362, 0.680)	(0.521, 0.776; 0.375, 0.703)
Sociodemographic	(0.582, 0.518; 0.272, 0.547)	(0.555, 0.542; 0.269, 0.558)	(0.510, 0.612; 0.277, 0.587)	(0.599, 0.558; 0.294, 0.601)	(0.541, 0.626; 0.298, 0.620)
Prenatal + Family History	(0.476, 0.786; 0.356, 0.639)	(0.517, 0.767; 0.366, 0.665)	(0.702, 0.666; 0.393, 0.734)	(0.688, 0.589; 0.345, 0.669)	(0.654, 0.713; 0.401, 0.742)
Prenatal + Sociodemographic	(0.442, 0.764; 0.320, 0.627)	(0.442, 0.769; 0.323, 0.631)	(0.589, 0.672; 0.344, 0.655)	(0.534, 0.650; 0.306, 0.638)	(0.606, 0.684; 0.359, 0.687)
Family History + Sociodemographic	(0.421, 0.773; 0.313, 0.632)	(0.589, 0.679; 0.348, 0.659)	(0.548, 0.754; 0.375, 0.702)	(0.630, 0.632; 0.341, 0.656)	(0.555, 0.762; 0.384, 0.723)
Prenatal + Family History + Sociodemographic	(0.630, 0.601; 0.326, 0.658)	(0.630, 0.669; 0.361, 0.695)	(0.664, 0.688; 0.390, 0.723)	(0.342, 0.866; 0.327, 0.682)	(0.668, 0.704; 0.402, 0.745)

**Fig. 1:**
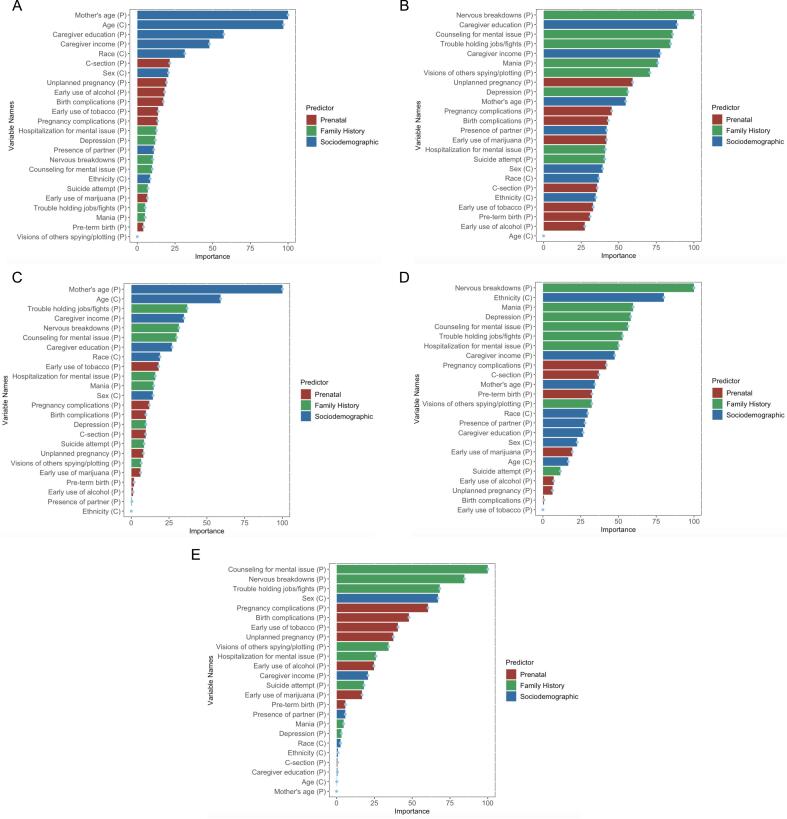
Relative variable importance for the Bagged CART (A), Random Forests (B), Gradient Boosted Trees (C), Neural Networks (D), and Elastic Net (E) ML models containing all 3 sets of predictors (variables denoted as P=Parental and C=Child).

All 5 ML algorithms differed in their ranking of different risk predictors (Fig. 1). The most important early life factors were ranked higher in at least 4 out of 5 ML models, including counseling for an emotional/mental issue (1B: 85.8 %, 1C: 30.0 %, 1D: 56.3 %, 1E: 100.0 %), nervous breakdowns (1B: 100.0 %, 1C: 31.5 %, 1D: 100.0 %, 1E: 84.6 %), and trouble holding jobs/fights (1B: 84.5 %, 1C: 37.0 %, 1D: 52.7 %, 1E: 68.2 %). Apart from elastic net, maternal age (1 A: 100.0 %, 1B: 54.6 %, 1C: 100.0 %, 1D: 34.4 %), caregiver income (1 A: 47.8 %, 1B: 77.6 %, 1C: 34.8 %, 1D: 47.5 %), and caregiver education (1 A: 57.4 %, 1B: 88.8 %, 1C: 27.0 %, 1D: 26.7 %) were also ranked as significant predictors of high CBCL scores. Child's age (1 A: 96.9 %, 1C: 59.1 %), sex (1 A: 20.7 %, 1B; 39.3 %, 1D: 22.7 %, 1E: 67.1 %), along with unplanned pregnancy (1 A: 19.4 %, 1B; 59.3 %, 1E: 37.5 %), pregnancy complications (1B; 45.3 %, 1D: 42.0 %, 1E: 60.5 %), birth complications (1B: 43.0 %, 1D: 42.0 %, 1E: 47.8 %) and parental visions of other spying/plotting (1B: 70.7 %, 1D: 32.3 %, 1E: 34.3 %), were ranked as important in at least 3 out of 5 ML models. Other risk factors such as depression, mania, and early use of tobacco were among the most important predictors in 2 out of 5 ML models.

## Discussion

### Main findings

This study employed both logistic regression and ML analysis to identify whether certain prenatal, family history, or sociodemographic risk factors were associated with clinically significant psychopathology among 9643 children in the ABCD Study. Our results indicate that early life exposures are independently linked with increased odds of high CBCL scores. When controlling for sociodemographic covariates, most exposures exhibited moderate reductions in these odds, exempting pregnancy complications, birth complications, pre-term birth and counseling for an emotional/mental issue. ML model performance was generally comparable across the 5 different algorithms, although there were notable differences. The bagged CART and gradient boosted trees models placed more emphasis on sociodemographic risk factors like maternal age, child's age, caregiver income and caregiver education. The random forests and neural networks relied on both sociodemographic factors and family history (e.g., trouble holding jobs/fights, nervous breakdowns, counseling for an emotional/mental issue, mania and depression). While elastic net prioritized prenatal and family history predictors, its higher performance was comparable to that of gradient boosted trees. The most important early life predictors were parental counseling for an emotional/mental issue, nervous breakdowns, and trouble holding jobs/fights. Although ML model performance generally improved when all 3 combinations of predictors were pooled together, the relative improvement of combining sociodemographic risk factors with prenatal and family history risk factors was modest at best.

### Comparison with other studies

Our study builds upon previous research examining the impact of early life exposures on adolescent psychopathology. Although prior studies have considered risk factors in isolation [[84], [85], [86]], few have attempted to examine the relative importance of these factors through a ML framework [15,18,19]. A recent study by Rothenberg et al. attempts to resolve this issue by ranking a wider range of predictors than those included in our analyses [15]. However, Rothenberg et al. also uses a comparatively smaller population of adolescents in their study [15]. Prenatal alcohol use and obstetric complications been noted previously for their influence on the development of mental health issues in children [[87], [88], [89], [90]]. On the contrary, few studies have investigated the impact of unplanned pregnancy and trouble holding jobs/fights on adolescent psychopathology [91,92]. To our knowledge, no studies have separately investigated the role of parental nervous breakdowns on adolescent mental health. Finally, the significance of sociodemographic factors may be understated in this study due to known differences between parental and youth reports of adolescent psychopathology [93,94].

### Strengths and limitations

The main strength of this study is the ML modeling in a comparatively large sample size relative to prior studies [15,95]. Since this example was intended to be didactic, we did not consider the effects of additional covariates such as genetics [96], extended family history (i.e., beyond parental background) [12], and the recent COVID-19 pandemic [97,98]. As our tutorial was created using R packages, we recognize that our recommendations may require additional programming to put in place on other platforms like scikit-learn in Python. It is also known that ML predictions within certain samples may not generalize well across other sample populations, thus validation of prediction models across different samples is necessary [99]. Future studies examining a wider range of ML algorithms and risk factors can further reduce prediction error.

### Implications and recommendations

Our study suggests that ML models incorporating early life factors could perhaps one day be used to guide clinical predictions of adolescent psychopathology. The elevated importance of family genetics and prenatal factors relative to social environment suggests that family history will play a critical role in producing accurate clinical predictions. A balanced approach between traditional screening programs and ML may provide the best results for monitoring at-risk adolescents at an early stage. It may be necessary to prioritize ML models with high sensitivity to minimize the rate of false negatives (i.e. undetected at-risk adolescents), even if this results in more false positives (i.e. lower specificity) as more adolescents would be screened for adverse psychiatric outcomes [100].

The modest predictive power of early life risk factors demonstrates the complexity of the ML model training and tuning process and suggests either that current ML approaches for predicting adolescent psychopathology may be inadequate, and/or that early life factors have limited ability to predict adolescent psychopathology. Incorporation of additional information such as genetics and more temporally proximal data such as adverse childhood experiences may help provide a more accurate adolescent risk classification system.

Standard regression models can offer insights into the individual influences of risk predictors and is recommended for use prior to the implementation of ML analysis. This method avoids the pitfalls associated with obtaining high-performing models that fail to explain their results due to the “black box” nature of ML [101]. The use of different random seeds can have inflationary effects on the performance of predictive models [102]. Due to the experimentation involved with ML and the potential risks associated with altering the system's default seed during each run [68], we suggest specifying a single random seed before training, testing, and validation stages of ML analysis. The choice of manual versus automatic tuning was not significant during our model training and will vary based on the researcher's preference and prediction outcome. When documenting the results of ML analyses, researchers should follow standard prediction model reporting guidelines (e.g. TRIPOD) and guidelines more specifically catered for ML [47,103].

## Conclusion

In summary, this study leverages data from 9643 adolescents in the ABCD Study to determine whether prenatal, family history, and sociodemographic factors play a significant role in predicting adolescent psychopathology. Our results indicate that although these early life factors may be associated with increased odds for developing clinically significant illness, the impact of other risk predictors cannot be overlooked. These findings suggest that ML one day may be useful for clinical risk prediction of adolescent psychopathology and or for informing interventions. Our study also offers suggestions and clarifies common complications associated with ML training, testing, and validation processes.

## Disclosures

None.

## CRediT authorship contribution statement

**Faizaan Siddique:** Writing – review & editing, Writing – original draft, Visualization, Software, Methodology, Investigation, Formal analysis. **Brian K. Lee:** Writing – review & editing, Supervision, Software, Resources, Project administration, Methodology, Investigation, Formal analysis.

## Declaration of competing interest

The authors declare no potential conflicts of interest.

## Appendix A Supplementary data

Supplementary material 1 Image 1

Supplementary material 2 Image 2

Supplementary material 3 Image 3

## References

[1] Kutcher S., Venn D. (2008). Why youth mental health is so important. Medscape J Med.

[2] Whitney D.G., Peterson M.D. (2019). US national and state-level prevalence of mental health disorders and disparities of mental health care use in children. JAMA Pediatr.

[3] Defar S., Abraham Y., Reta Y. (2023). Health related quality of life among people with mental illness: the role of socio-clinical characteristics and level of functional disability. Front Public Health.

[4] Galván A. (2014). Insights about adolescent behavior, plasticity, and policy from neuroscience research. Neuron.

[5] Essau C.A., Sasagawa S., Lewinsohn P.M., Rohde P. (2018). The impact of pre- and perinatal factors on psychopathology in adulthood. J Affect Disord.

[6] Zaman R., Hankir A., Jemni M. (2019). Lifestyle factors and mental health. Psychiatr Danub.

[7] McGrath J.J., Wray N.R., Pedersen C.B., Mortensen P.B., Greve A.N., Petersen L. (2014). The association between family history of mental disorders and general cognitive ability. Transl Psychiatry.

[8] Campbell F., Blank L., Cantrell A. (2022). Factors that influence mental health of university and college students in the UK: a systematic review. BMC Public Health.

[9] Hales C.N., Barker D.J., Clark P.M. (1991). Fetal and infant growth and impaired glucose tolerance at age 64. BMJ.

[10] Barker D.J.P. (2007). The origins of the developmental origins theory. J Intern Med.

[11] Tearne J.E., Allen K.L., Herbison C.E. (2015). The association between prenatal environment and children’s mental health trajectories from 2 to 14 years. Eur Child Adolesc Psychiatry.

[12] Zuckerman B., Wong S.L. (2019). Family history: an opportunity to disrupt transmission of behavioral health problems. Pediatrics.

[13] Roffman J.L., Sipahi E.D., Dowling K.F. (2021). Association of adverse prenatal exposure burden with child psychopathology in the adolescent brain cognitive development (ABCD) study. PloS One.

[14] Tsuang M.T., Bar J.L., Stone W.S., Faraone S.V. (2004). Gene-environment interactions in mental disorders. World Psychiatry.

[15] Rothenberg W.A., Bizzego A., Esposito G. (2023). Predicting adolescent mental health outcomes across cultures: a machine learning approach. J Youth Adolesc.

[16] Dwyer D.B., Falkai P., Koutsouleris N. (2018). Machine learning approaches for clinical psychology and psychiatry. Annu Rev Clin Psychol.

[17] Iyortsuun N.K., Kim S.H., Jhon M., Yang H.J., Pant S. (2023). A review of machine learning and deep learning approaches on mental health diagnosis. Healthcare.

[18] Tate A.E., McCabe R.C., Larsson H., Lundström S., Lichtenstein P., Kuja-Halkola R. (2020). Predicting mental health problems in adolescence using machine learning techniques. PloS One.

[19] Dobias M.L., Sugarman M.B., Mullarkey M.C., Schleider J.L. (2022). Predicting mental health treatment access among adolescents with elevated depressive symptoms: machine learning approaches. Administration and Policy in Mental Health and Mental Health Services Research.

[20] Chung J., Teo J. (2022). Mental health prediction using machine learning: taxonomy, applications, and challenges. Applied Computational Intelligence and Soft Computing.

[21] Vabalas A., Gowen E., Poliakoff E., Casson A.J. (2019). Machine learning algorithm validation with a limited sample size. PloS One.

[22] Wiemken T.L., Kelley R.R. (2020). Machine learning in epidemiology and health outcomes research. Annu Rev Public Health.

[23] Bellman R. (1966). Dynamic programming. Science (1979).

[24] Hu Z., Shukla K., Karniadakis G.E., Kawaguchi K. (2024). Tackling the curse of dimensionality with physics-informed neural networks. Neural Netw.

[25] Banks D.L., Fienberg S.E. (2003).

[26] Chen Z.S., Kulkarni P (Param), Galatzer-Levy I.R., Bigio B., Nasca C., Zhang Y. (2022). Modern views of machine learning for precision psychiatry. Patterns.

[27] Obermeyer Z., Emanuel E.J. (2016). Predicting the future — big data, machine learning, and clinical medicine. N Engl J Med.

[28] Fernandes B.S., Williams L.M., Steiner J., Leboyer M., Carvalho A.F., Berk M. (2017). The new field of ‘precision psychiatry’. BMC Med.

[29] Zhou Z., Wu T.C., Wang B., Wang H., Tu X.M., Feng C. (2020). Machine learning methods in psychiatry: a brief introduction. Gen Psychiatr.

[30] Shatte A.B.R., Hutchinson D.M., Teague S.J. (2019). Machine learning in mental health: a scoping review of methods and applications. Psychol Med.

[31] Jiang T., Gradus J.L., Rosellini A.J. (2020). Supervised machine learning: a brief primer. Behav Ther.

[32] Pike A.C., Robinson O.J. (2022). Reinforcement learning in patients with mood and anxiety disorders vs control individuals. JAMA Psychiatry.

[33] Koppe G., Meyer-Lindenberg A., Durstewitz D. (2021). Deep learning for small and big data in psychiatry. Neuropsychopharmacology.

[34] Shen D., Wu G., Suk H.I. (2017). Deep learning in medical image analysis. Annu Rev Biomed Eng.

[35] Quaak M., van de Mortel L., Thomas R.M., van Wingen G. (2021). Deep learning applications for the classification of psychiatric disorders using neuroimaging data: systematic review and meta-analysis. Neuroimage Clin.

[36] Zhao K., Duka B., Xie H., Oathes D.J., Calhoun V., Zhang Y. (2022). A dynamic graph convolutional neural network framework reveals new insights into connectome dysfunctions in ADHD. Neuroimage.

[37] Khosla M., Jamison K., Kuceyeski A., Sabuncu M.R. (2019). Ensemble learning with 3D convolutional neural networks for functional connectome-based prediction. Neuroimage.

[38] Zhu H., Yuan M., Qiu C. (2020). Multivariate classification of earthquake survivors with post-traumatic stress disorder based on large-scale brain networks. Acta Psychiatr Scand.

[39] Watson D.S., Krutzinna J., Bruce I.N. (2019). Clinical applications of machine learning algorithms: beyond the black box. BMJ.

[40] Karcher N.R., Barch D.M. (2021). The ABCD study: understanding the development of risk for mental and physical health outcomes. Neuropsychopharmacology.

[41] Iacono W.G., Heath A.C., Hewitt J.K. (2018). The utility of twins in developmental cognitive neuroscience research: how twins strengthen the ABCD research design. Dev Cogn Neurosci.

[42] Jernigan T.L., Brown S.A. (2018). Introduction. Dev Cogn Neurosci.

[43] Karcher N.R., Barch D.M., Avenevoli S. (2018). Assessment of the prodromal questionnaire-brief child version for measurement of self-reported Psychoticlike experiences in childhood. JAMA Psychiatry.

[44] Auchter A.M., Hernandez Mejia M., Heyser C.J. (2018). A description of the ABCD organizational structure and communication framework. Dev Cogn Neurosci.

[45] Menken M.S., Isaiah A., Liang H. (2022). Peer victimization (bullying) on mental health, behavioral problems, cognition, and academic performance in preadolescent children in the ABCD study. Front Psychol.

[46] Cuschieri S. (2019). The STROBE guidelines. Saudi J Anaesth.

[47] Collins G.S., Reitsma J.B., Altman D.G., Moons K. (2015). Transparent reporting of a multivariable prediction model for individual prognosis or diagnosis (TRIPOD): the TRIPOD statement. BMC Med.

[48] Achenbach T.M., Rescorla L.A. (2000).

[49] Mazefsky C.A., Anderson R., Conner C.M., Minshew N. (2011). Child behavior checklist scores for school-aged children with autism: preliminary evidence of patterns suggesting the need for referral. J Psychopathol Behav Assess.

[50] Ford S.H., McCoy T.P. (2022). Minding the gap: adolescent and parent/caregiver reporter discrepancies on symptom presence, impact of covariates, and clinical implications. J Pediatr Health Care.

[51] Wen X., Shu Y., Qu D. (2023). Associations of bullying perpetration and peer victimization subtypes with preadolescent’s suicidality, non-suicidal self-injury, neurocognition, and brain development. BMC Med.

[52] Karcher N.R., Klaunig M.J., Elsayed N.M., Taylor R.L., Jay S.Y., Schiffman J. (2022). Understanding associations between race/ethnicity, experiences of discrimination, and psychotic-like experiences in middle childhood. J Am Acad Child Adolesc Psychiatry.

[53] Pan S., Chen S. (2023). Empirical comparison of imputation methods for multivariate missing data in public health. Int J Environ Res Public Health.

[54] Nijman S., Leeuwenberg A., Beekers I. (2022). Missing data is poorly handled and reported in prediction model studies using machine learning: a literature review. J Clin Epidemiol.

[55] Jäger S., Allhorn A., Bießmann F. (2021). A benchmark for data imputation methods. Front Big Data.

[56] Raghunathan T.E., Solenberger P.W., Van Hoewyk J. (2002). IVEware: imputation and variance estimation software user guide IVEware. Imputation and Variance Estimation Software.

[57] Ghasemi M., Samadi M., Soleimanian E., Chau K.W. (2023). A comparative study of black-box and white-box data-driven methods to predict landfill leachate permeability. Environ Monit Assess.

[58] Sayed B.T., Al-Mohair H.K., Alkhayyat A., Ramírez-Coronel A.A., Elsahabi M. (2023). Comparing machine-learning-based black box techniques and white box models to predict rainfall-runoff in a northern area of Iraq, the little Khabur River. Water Sci Technol.

[59] Tripepi G., Jager K.J., Dekker F.W., Zoccali C. (2008). Linear and logistic regression analysis. Kidney Int.

[60] Sarker I.H. (2021). Machine learning: algorithms, real-world applications and research directions. SN Comput Sci.

[61] Kuhn M. (2008). Building predictive models in *R* using the **caret** package. J Stat Softw.

[62] Ahmed A., Sultana R., Ullas M.T.R., Begom M., Rahi MdMI, MdA Alam (2020). IEEE Asia-Pacific conference on computer science and data engineering (CSDE).

[63] Vergyri D., Knoth B., Shriberg E. (2015). Sixteenth Annual Conference of the International Speech Communication Association.

[64] Miller M.I., Shih L.C., Kolachalama V.B. (2023). Machine learning in clinical trials: a primer with applications to neurology. Neurotherapeutics.

[65] Kumar Dhairya (2024). Introduction to data preprocessing in machine learning | by Dhairya Kumar | towards data science. Towards Data Science Published December 25, 2018. https://towardsdatascience.com/introduction-to-data-preprocessing-in-machine-learning-a9fa83a5dc9d.

[66] Subramanian J., Simon R. (2013). Overfitting in prediction models – is it a problem only in high dimensions?. Contemp Clin Trials.

[67] Gholamy A., Kreinovich V., Kosheleva O. (2018). Why 70/30 or 80/20 Relation Between Training and Testing Sets: A Pedagogical Explanation. https://scholarworks.utep.edu/cs_techrep/1209.

[68] Beam A.L., Manrai A.K., Ghassemi M. (2020). Challenges to the reproducibility of machine learning models in health care. JAMA.

[69] Bischl B., Mersmann O., Trautmann H., Weihs C. (2012). Resampling methods for Meta-model validation with recommendations for evolutionary computation. Evol Comput.

[70] Tougui I., Jilbab A., El Mhamdi J. (2021). Impact of the choice of cross-validation techniques on the results of machine learning-based diagnostic applications. Healthc Inform Res.

[71] Dinov I.D., Christou N., Gould R. (2009). Law of large numbers: the theory, applications and technology-based education. Journal of Statistics Education.

[72] Walters S.J., Campbell M.J. (2004). The use of bootstrap methods for analysing health-related quality of life outcomes (particularly the SF-36). Health Qual Life Outcomes.

[73] Pfob A., Lu S.C., Sidey-Gibbons C. (2022). Machine learning in medicine: a practical introduction to techniques for data pre-processing, hyperparameter tuning, and model comparison. BMC Med Res Methodol.

[74] Pfob A., Lu S.C., Sidey-Gibbons C. (2022). Machine learning in medicine: a practical introduction to techniques for data pre-processing, hyperparameter tuning, and model comparison. BMC Med Res Methodol.

[75] Wang Q., Ma Y., Zhao K., Tian Y. (2022). A comprehensive survey of loss functions in machine learning. Annals of Data Science.

[76] Hicks S.A., Strümke I., Thambawita V. (2022). On evaluation metrics for medical applications of artificial intelligence. Sci Rep.

[77] Adhikari S., Normand S.L., Bloom J., Shahian D., Rose S. (2021). Revisiting performance metrics for prediction with rare outcomes. Stat Methods Med Res.

[78] Müller D., Soto-Rey I., Kramer F. (2022). Towards a guideline for evaluation metrics in medical image segmentation. BMC Res Notes.

[79] Rainio O., Teuho J., Klén R. (2024). Evaluation metrics and statistical tests for machine learning. Sci Rep.

[80] Esposito C., Landrum G.A., Schneider N., Stiefl N., Riniker S. (2021). GHOST: adjusting the decision threshold to handle imbalanced data in machine learning. J Chem Inf Model.

[81] Ruopp M.D., Perkins N.J., Whitcomb B.W., Schisterman E.F. (2008). Youden index and optimal cut-point estimated from observations affected by a lower limit of detection. Biom J.

[82] Thiele C., Hirschfeld G. (2021). **Cutpointr** : improved estimation and validation of optimal Cutpoints in *R*. J Stat Softw.

[83] Šimundić A.M. (2009). Measures of diagnostic accuracy: basic definitions. EJIFCC.

[84] Eilertsen E.M., Gjerde L.C., Reichborn-Kjennerud T. (2017). Maternal alcohol use during pregnancy and offspring attention-deficit hyperactivity disorder (ADHD): a prospective sibling control study. Int J Epidemiol.

[85] Behere A.P., Basnet P., Campbell P. (2017). Effects of family structure on mental health of children: a preliminary study. Indian J Psychol Med.

[86] Afroz N., Kabir E., Alam K. (2023). A latent class analysis of the socio-demographic factors and associations with mental and behavioral disorders among Australian children and adolescents. PloS One.

[87] Verdoux H., Sutter A.L. (2002). Perinatal risk factors for schizophrenia: diagnostic specificity and relationships with maternal psychopathology. Am J Med Genet.

[88] Eaton W.W., Mortensen P.B., Thomsen P.H., Frydenberg M. (2001). Obstetric complications and risk for severe psychopathology in childhood. J Autism Dev Disord.

[89] Larkby C.A., Goldschmidt L., Hanusa B.H., Day N.L. (2011). Prenatal alcohol exposure is associated with conduct disorder in adolescence: findings from a birth cohort. J Am Acad Child Adolesc Psychiatry.

[90] Staroselsky A., Fantus E., Sussman R., Sandor P., Koren G., Nulman I. (2009). Both parental psychopathology and prenatal maternal alcohol dependency can predict the behavioral phenotype in children. Pediatric Drugs.

[91] Myhrman A., Rantakallio P., Isohanni M., Jones P., Partanen U. (1996). Unwantedness of a pregnancy and schizophrenia in the child. Br J Psychiatry.

[92] Moustgaard H., Avendano M., Martikainen P. (2018). Parental unemployment and offspring psychotropic medication purchases: a longitudinal fixed-effects analysis of 138,644 adolescents. Am J Epidemiol.

[93] Chen Y.Y., Ho S.Y., Lee P.C., Wu C.K., Gau S.S.F. (2017). Parent-child discrepancies in the report of adolescent emotional and behavioral problems in Taiwan. PloS One.

[94] Robinson M., Doherty D.A., Cannon J. (2019). Comparing adolescent and parent reports of externalizing problems: a longitudinal population-based study. Br J Dev Psychol.

[95] Sumathi Ms B., Dr (2016). Prediction of mental health problems among children using machine learning techniques. International Journal of Advanced Computer Science and Applications.

[96] Lee P.H., Doyle A.E., Li X. (2022). Genetic Association of Attention-Deficit/hyperactivity disorder and major depression with suicidal ideation and attempts in children: the adolescent brain cognitive development study. Biol Psychiatry.

[97] Caffo E., Asta L., Scandroglio F. (2021). Predictors of mental health worsening among children and adolescents during the coronavirus disease 2019 pandemic. Curr Opin Psychiatry.

[98] Zhou S.J., Zhang L.G., Wang L.L. (2020). Prevalence and socio-demographic correlates of psychological health problems in Chinese adolescents during the outbreak of COVID-19. Eur Child Adolesc Psychiatry.

[99] Yang J., Soltan A.A.S., Clifton D.A. (2022). Machine learning generalizability across healthcare settings: insights from multi-site COVID-19 screening. NPJ Digit Med.

[100] Uchida M., Bukhari Q., DiSalvo M. (2022). Can machine learning identify childhood characteristics that predict future development of bipolar disorder a decade later?. J Psychiatr Res.

[101] de Lacy N., Ramshaw M.J., McCauley E., Kerr K.F., Kaufman J., Nathan Kutz J. (2023). Predicting individual cases of major adolescent psychiatric conditions with artificial intelligence. Transl Psychiatry.

[102] Henderson P., Islam R., Bachman P., Pineau J., Precup D., Meger D. (2018). Deep Reinforcement Learning That Matters. Proceedings of the AAAI Conference on Artificial Intelligence.

[103] Stevens L.M., Mortazavi B.J., Deo R.C., Curtis L., Kao D.P. (2020). Recommendations for reporting machine learning analyses in clinical research. Circ Cardiovasc Qual Outcomes.

[104] Lin E., Lin C.H., Lane H.Y. (2021). Applying a bagging ensemble machine learning approach to predict functional outcome of schizophrenia with clinical symptoms and cognitive functions. Sci Rep.

[105] Li Y., Zhang L., Zhang Y. (2021). A random Forest model for predicting social functional improvement in Chinese patients with schizophrenia after 3 months of atypical antipsychotic Monopharmacy: a cohort study. Neuropsychiatr Dis Treat.

[106] Ali F.Z., Wengler K., He X., Nguyen M.H., Parsey R.V., DeLorenzo C. (2022). Gradient boosting decision-tree-based algorithm with neuroimaging for personalized treatment in depression. Neuroscience Informatics.

[107] Uyulan C., Ergüzel T.T., Unubol H. (2021). Major depressive disorder classification based on different convolutional neural network models: deep learning approach. Clin EEG Neurosci.

[108] Kim M.H., Banerjee S., Park S.M., Pathak J. (2016). Improving risk prediction for depression via elastic net regression - Results from Korea National Health Insurance Services Data. AMIA Annu Symp Proc.

